# Hair Mercury Negatively Correlates with Calcium Pump Activity in Human Term Newborns and Their Mothers at Delivery

**DOI:** 10.1289/ehp.10381

**Published:** 2007-11-17

**Authors:** Guy Huel, Josiane Sahuquillo, Ginette Debotte, Jean-François Oury, Larissa Takser

**Affiliations:** 1 Institut National de la Santé et de la Recherche Médicale (INSERM-IFR69-U780), Recherche en Épidemiologie et en Biostatistique, Villejuif, France; 2 Département d’Obstétrique et Gynécologie, Hôpital Robert Debré, Paris, France; 3 Département d’Obstétrique et Gynécologie, Faculté de Médecine, Université de Sherbrooke, Québec, Canada

**Keywords:** calcium, cord blood, environmental exposure, lead, mercury, newborn, pregnancy

## Abstract

**Background:**

Calcium homeostasis is a known target of several environmental toxicants including lead and mercury.

**Objective:**

Our goal was to determine the relationship between Hg exposure and erythrocyte Ca pump activity in women at delivery and in their newborns.

**Methods:**

We determined total Hg as well as Pb concentrations in 81 hair and blood samples obtained at delivery. Basal and calmodulin-stimulated Ca pump activity was measured in red blood cells from cord blood and maternal erythrocyte plasma membranes.

**Results:**

Maternal hair Hg negatively correlates with Ca pump activity in maternal and cord blood erythrocytes. Pb and Hg both independently correlate negatively with Ca pump activity without any statistically significant interaction. After adjustment for potential confounders, Pb and Hg explain about 30% and 7% of total variance of Ca pump activity in newborns and mothers, respectively.

**Conclusion:**

Our findings confirm results reported in previous experimental studies and support the use of biomarkers in newborns from general population.

The neurotoxic effects of prenatal exposure to high doses of methylmercury (MeHg) were well established after the serious poisoning epidemics in Japan and Iraq (Harada 1995; Marsh et al. 1987). However, associations between MeHg exposure, developmental milestones, and neurologic tests at exposure levels as low as 10 ppm (estimated in maternal hair) have proven difficult to confirm (Myers and Davidson 2000). Nevertheless, children exposed to mercury levels < 10 ppm have been observed to display subtle neurobehavioral deficits, especially with respect to language, attention, and memory scores (Grandjean et al. 1997; Weihe et al. 2002). In one of three main cohorts, beneficial associations observed between Hg exposure and psychomotor performances (Myers et al. 1995) are believed to be attributable to other nutriments found in fish, such as selenium and/or polyunsaturated fatty acids. Although high-dose MeHg poisoning has been shown to cause developmental disorders, evidence that more subtle low-level developmental exposure contributes to these disorders is limited.

Sensitive biomarkers are needed to assess the effects consequent to low-dose exposures. Calcium homeostasis is a known target of several environmental neurotoxicants including lead and Hg (Habermann et al. 1983; Kirk et al. 2003; Marty and Atchison 1997). Deregulation of Ca homeostasis triggers serious effects on cell functioning because of altered Ca signaling. Normally, cytosolic concentration of free Ca^2+^ (divalent calcium cation) is maintained at low levels (10^−7^ M) by extrusion and compartmentalization systems. A main component of the extrusion process is the plasma membrane Ca–ATPase pump (Carafoli and Brini 2000). Whereas transient elevations of cytosolic Ca^2+^ levels are an important component of cell signaling, sustained Ca^2+^ increases are not tolerated by cells and generally lead to apoptosis or cytotoxicity and necrosis (Orrenius et al. 1989). Intracellular Ca is a sensitive biomarker of pollutant-related stress in amoebic cells exposed to Hg at sub-lethal doses (Dondero et al. 2006).

Heavy metals are able to inhibit *in vitro* Ca pump activity in purified plasma membranes obtained from mussel-gill cells. Experimental studies have shown that Hg^2+^ inhibits Ca pump activity by preventing the formation of an essential phosphorylated intermediate of the process of Ca^2+^ transport (Viarengo et al. 1993). One *in vitro* study reported the compensatory activation of Ca pump protein expression after Hg induced transitory inhibition of Ca pump activity, leading to a net increase in enzyme activity (Burlando et al. 2004).

We previously reported a negative association between low prenatal Pb exposure and red-blood-cell Ca pump activity in healthy newborns (Campagna et al. 2000). Because prenatal Hg exposure at high levels induces widespread damage to the developing brain, we assumed that more subtle low-level exposures should trigger more subtle functional disturbances. Accordingly, we hypothesized that Ca pump activity would also be correlated with low-level prenatal Hg exposure. Thus, our objective was to determine the relationships between Hg and Pb prenatal exposures and erythrocyte Ca pump activity in human term newborns and their mothers at delivery. We used Hg content in maternal hair as a biomarker of Hg exposure, which was shown to be highly predictive for MeHg in maternal and cord blood in low-fish-eating pregnant populations (Morrissette et al. 2004).

## Materials and Methods

### Subjects

Ninety-eight mother–newborn pairs were recruited at Robert Debré Maternity Hospital (Paris, France). This large pediatric hospital predominantly services suburban and northeastern parts of Paris. All mothers provided informed consent before study participation. Obstetric history was obtained from medical records for the following exclusion criteria: stillbirths, multiple births, congenital malformations, pregnancies under regular drug treatment (for diabetes, hypertension, asthma, hypothyroidism, or other chronic diseases), and births with cesarean sections or at a gestational age < 37 weeks. A standardized questionnaire was administered to study mothers by the same observer on the third day after delivery. The questionnaire detailed the daily average number of cigarettes smoked, daily intake of tap water, coffee, and tea, and weekly consumption of wine, beer, cider, and liquors. Study mothers were classified as smokers if they smoked at least one cigarette per day, as alcohol drinkers if they drank at least one alcoholic beverage per week on a regular basis throughout their pregnancy, and as coffee or tea drinkers if they drank at least one cup of tea or coffee daily throughout their pregnancy.

### Sampling and biochemical analyses

#### Blood and hair collection

We sampled maternal and cord blood at delivery in heparinized Vacutainer tubes and stored at 4°C until analysis. Hair samples were taken from the occipital region of the head. All hair samples weighed > 10 mg. Hair 3 cm from the root was discarded. Average hair grows about 1 cm/month, with a latency period of 1 month between toxicant exposure and appearance of toxicant-exposed hair at the scalp surface. Thus, hair samples collected at delivery reflected exposures during the 3–4 months preceding the final month of pregnancy.

#### Erythrocyte plasma membrane preparation

We prepared the plasma membrane within 20 hr after blood withdrawal. Red blood cell (ghost) membrane suspensions were obtained according to a method modified from Hanahan and Ekholm (1974). Samples were centrifuged at 1,000 × *g* for 15 min at 4°C. Plasma and buffy coat were removed by careful suction, and cells were re-suspended in isotonic Tris–HCl buffer (pH 6.0). After mixing by inversion, samples were recentrifuged at 1,000 × *g* for 15 min at 4°C. Supernatant was removed by careful suction, and a few red cells were sacrificed to remove any remaining buffy layer. This washing procedure was repeated twice. Washed cells were suspended in isotonic Tris–HCl buffer (pH 7.4) to an approximate hematocrit of 50% and then kept on ice. Samples were mixed gently by inversion for approximately 1 minute before plasma membrane preparation.

We transferred 5-mL aliquots of the above 50% cell suspensions to 50-mL polyethylene tubes. We added 30 mL hypotonic Tris–HCl buffer (pH 7.6) to the cell suspension for osmotic lysis. Tubes were allowed to stand for approximately 10 min before centrifugation at 15,000 × *g* for 20 min. Supernatants were discarded and pellets were resuspended in 8 mL of Tris–HCl (pH 7.4) and centrifuged for 20 min at 15,000 × *g*. Pellets were then washed four times until membranes appeared colorless to the naked eye. The last colorless pellet was then rinsed twice with 50 μL cold Tris–HCl (pH 7.4) and poured into a cryotube. Membrane suspensions were kept frozen in the latter buffer at −80°C until further analysis.

#### Determination of Pb and total Hg concentrations

We measured Pb concentrations in maternal and cord blood as well as in maternal and newborn hair by flameless atomic absorption spectrophotometry using a Zeeman effect corrector (PerkinElmer 4100 ZL; PerkinElmer, Courtaboeuf, France). Accuracy and reliability of Pb determination were checked by national evaluation of quality control: The quality is checked every 3 months by determining the level of Pb in samples sent to the Agence Française de Sécurité des Médicaments (French Agency for Drugs Safety). Methods used for sampling, preparation of hair samples, and chemical analysis are described extensively elsewhere (Huel et al. 1984).

Hair Hg concentration was determined in maternal hair by cold vapor atomic absorption (Perkin Elmer AA 600). Before analyses, hair samples were digested with nitric acid for 2 hr at 70°C, and then oxidized with potassium permanganate at 90°C for 15 min. Pb and Hg concentrations in hair are expressed in micrograms per gram. Pb in blood is expressed in micrograms per liter.

#### Determination of basal and calmodulin-stimulated Ca pump activities

We measured Ca pump activity using a modification of the method described by Beutler et al. (1983). Enzymatic activity reflected the amount of inorganic phosphate (Pi) produced by adenosine triphosphate (ATP) hydrolysis. Erythrocyte plasma membrane dilution were prepared in 5-mL tubes by adding 400 μL of unfrozen membrane suspension to 3 mL of basic medium composed of TrisHCl 92 mM (pH 7.4), MgCl2 5 mM, NaCl 100 mM, EGTA and water. We prepared three reaction tubes (A, B, and C) containing 2.5 mL of basic medium and 500 μL of plasma membrane dilution. We added volumes of 150 μL Tris–HCl (pH 7.4) to tube A; 140 μL Tris–HCl and 10 μL CaCl2 to tube B; and 130 μL Tris–HCl, 10 μL CaCl2, and 10 μL of calmodulin to tube C. Reactions were initiated by adding 50 μL ATP. Tubes were then incubated for 30 min at 37°C, Reactions were stopped by addition of 500 μL of trichloroacetic acid 5%. Tubes were then centrifuged at 4°C for 10 min at 4,000 × *g*. We performed colorimetric assessment according to the Hurst method (Hurst 1964). Analyses were performed in triplicate. We calculated basal Ca pump activity as the activity difference between tubes B and A. We calculated calmodulin-stimulated Ca pump activity as the activity difference between tubes C and A.

We measured protein concentrations of plasma membrane preparations with a Biorad Protein Assay Kit (Bio-Rad Laboratories GmbH, Heidemannitiabe, Germany) using bovine serum albumin (BSA) as a standard. All results were corrected for reagent blank and calculated as the mean of two measurements. Activity is expressed in nanomoles of inorganic phosphate produced per milligram protein per hour (nmol/mg/hr). Measurement error (σ) was estimated between 278 and 348 nmol/mg/hr. Chemicals were of analytical grade (ATP and BSA: Sigma, St. Louis, MO, USA; others: Merck, Darmstadt, Germany).

### Statistical analyses

We performed statistical analyses using SAS version 9.1 (SAS Institute Inc., Cary, NC, USA). Because of skewed distribution, maternal hair Hg and PB (HgHM and PbHM), newborn hair Pb (PbHC), maternal and cord blood Pb (PbBM and PbBC), maternal and newborn plasma membrane Ca pump basal activities (CaM and CaC), and maternal and newborn membrane Ca pump calmodulin-stimulated activities (CaMc and CaCc) were transformed to their logarithms. Potential confounders considered for the relationship between enzyme activities and HgHM, PbHM, PbHC, PbBM, PbBC were maternal and paternal age at birth, maternal height and weight, gestational age, duration of labor and delivery, newborn height, cranial perimeter, birth weight, parity, newborn sex, maternal and paternal ethnicity, parental educational level, smoking and alcohol consumption during pregnancy, and coffee and tea consumption during pregnancy.

To isolate an optimal subset of potential confounders, we performed a forward stepwise linear regression analysis with confounders included as independent variables to explain maternal and cord Ca pump activities. Independent variables were entered one after the other, depending on their respective contribution to the explained variance, until the remaining factors failed to reach the 15% significance level of the *F* statistics. We followed a similar procedure to examine the relationship between these potential confounders and heavy metal levels. We performed separate analyses for maternal and newborn hair and blood Pb (PbHM, PbHC, PbBM, and PbBC).

Next, we analyzed relationships between Ca pump activities and heavy metal measurements via simple regression models. We then analyzed the relationship between Hg and Pb and Ca pump activities using multiple linear regression models. Models were designed with each measurement of Ca pump activity as the dependent variable. Hair Hg contents and blood or hair Pb levels were used as independent variables.

## Results

### Population and biological variables

Maternal mean (± SD) age at delivery was 29.7 ± 4.6 years. Forty-seven percent of study mothers were primiparous. Fifty-five percent of newborns were of male sex, mean birth weight was 3,403 ± 389 g, and gestational age was 39.8 ± 1.2 weeks.

Maternal Ca pump activities were obtained from only 81 samples, because of either lack of biological material or procedure failure. Therefore, complete data are available for 81 study subjects. Means and distributions for these 81 subjects are shown in Table 1.

### Predictive variables for Hg, Pb, and Ca pump activities

In linear regression models using a forward stepwise procedure including all potential confounders, only gestational age was related to Ca pump activities in study mothers (*p* = 0.14 and *p* = 0.10 for CaMc and CaM, respectively) and was also one of the considered predictive factors (*p* = 0.15) for newborn hair Pb. In the subsequent analysis, only newborn hair Pb was corrected for gestational age to avoid overmatching in the statistical analysis.

Ca pump activities in newborn blood were not related to the potential confounders considered. Thus, only gestational age (for models with Pb) and maternal Ca pump activity (to explain cord Ca pump activity) were introduced in multiple models.

### Correlations between maternal and newborn Ca pump activities

We observed positive and highly statistically significant links between Ca pump activities, mainly between basal and stimulated activities both in mothers and newborns (*r* = 0.95, *p* < 0.0001; and *r* = 0.98, *p* < 0.0001, respectively). To a lesser extent, Ca pump activities between mothers and newborns were also associated (*r* = 0.43, *p* < 0.0001 for basal activity; and *r* = 0.36, *p* < 0.0001 for stimulated activity).

### Correlation between Hg and Pb concentrations

Maternal hair Hg was not statistically correlated to any of the Pb indicators considered. Only maternal blood Pb and cord blood Pb levels were statistically correlated (*r* = 0.53; *p* < 0.0001).

### Relationships between Hg and Ca pump activities in simple linear regression models

**Figure 1:**
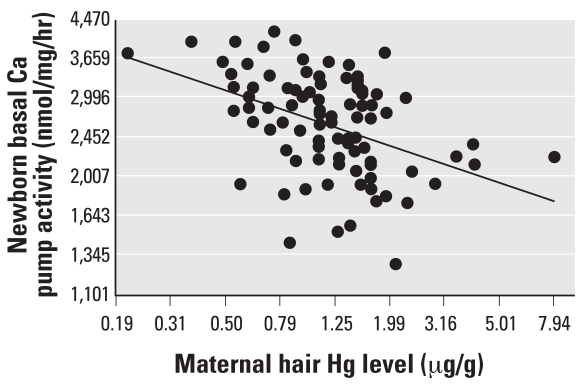
The association between the basal Ca pump activity in cord blood and the Hg concentration in maternal hair at delivery (*p* = 0.0002).

We constructed models with maternal or newborn basal or stimulated Ca pump activities as the dependent variable. Hair Hg was used as the independent variable. Simple regression analysis showed that Ca pump activities in mother—both basal and stimulated—negatively correlated with maternal hair Hg levels both for all maternal and cord blood (*r* = −0.36, *p* = 0.001; and *r* = −0.34, *p* = 0.002, respectively for mother; and *r* = −0.37, *p* = 0.0002; and *r* = −0.24, *p* = 0.01, respectively for cord blood). The simple regression plot for Ca pump activity in cord blood is shown in Figure 1.

### Relationships between Pb and Ca pump activities in simple linear regression models

Relationships between Ca pump activities and Pb measurements (as assessed both in hair and blood) were statistically significant only with basal Ca pump activity in cord blood (*p* < 0.01 for both). Maternal Pb concentrations did not correlate with Ca pump activities in maternal and cord blood.

### Multiple modeling to explain Ca pump activities in maternal blood

Maternal Ca pump activities significantly correlated with hair Hg concentrations (Tables 2 and 3), but not with any biomarker of Pb exposure.

### Multiple modeling to explain Ca pump activities in cord blood

No change occurred between Ca pump activities and maternal hair Hg levels when taking into account either maternal or newborn hair Pb contents or maternal or cord blood Pb contents. Cord basal Ca pump activity and newborn Pb exposure (as assessed by hair and blood Pb) maintained a significant statistical level when adjusted for Hg (Tables 2 and 3). There was no significant interaction between Hg and Pb. It is noteworthy that 33% of the newborn basal Ca pump activity variance was explained by maternal and newborn hair Hg (Table 2).

Adjustment of previously observed relationships with cord blood basal Ca pump activities for maternal Ca pump activities did not produce any substantial changes (Table 4). Three parameters—maternal hair Hg, maternal Ca pump basal activity, and newborn hair Pb—explained 40% of the newborn Ca pump basal activity variance.

For newborn stimulated Ca pump activity, the regression coefficient between newborn Ca pump calmodulin-stimulated activity and maternal hair Hg reached the 5% significance level, but maternal hair Hg correlated to newborn Ca pump calmodulin-stimulated activity to a lesser extent than observed with basal Ca pump activities (Table 4).

## Discussion

This study is the first to report the relationship between Ca pump activity in pregnant women and their newborns on the one hand and Hg exposure during pregnancy on the other hand. In newborns, both Hg and Pb strongly and independently negatively correlated to Ca pump activity in cord blood without any statistically significant interaction. In study mothers, only hair Hg significantly correlated to Ca pump activity. Moreover, whereas study mothers were exposed to very low levels of Hg and Pb compared with pregnant subjects of previously reported studies (Fok et al. 2007; Gibicar et al. 2006; Grandjean et al. 1999; Muckle et al. 2001; Myers et al. 1995; Pinheiro et al. 2005), exposure was nevertheless slightly higher than in other pregnant women studied in Québec (Canada) (Morrissette et al. 2004), the United States (Stern et al. 2001), and Sweden (Bjornberg et al. 2003). Maternal hair Hg in the Faroe study reported long-term adverse developmental effects of prenatal Hg exposure which was 5- to 10-fold higher than for our study group (Debes et al. 2006). Total Hg in hair correlates well with MeHg exposure and with brain levels in humans (Cernichiari et al. 1995). Thus, hair Hg measurements may provide an indirect and noninvasive method of measuring MeHg levels in the brain.

In our study, both hair and blood Pb negatively correlated to cord blood Ca pump activity. However, hair Pb was a better predictor of Ca pump activity than cord blood Pb. The known affinity of Pb, similar to MeHg, for thiol groups may thus explain accumulation of Pb in hair and its correlation with certain enzymatic activities. Hair Pb has been criticized as a biomarker of Pb exposure because of external contamination risks and high interlaboratory and within-individual variability (Barbosa et al. 2005). Newborn hair should not be at risk of external contamination because it is in contact with amniotic fluid only. Thus newborn hair Pb reflects both Pb accumulation and elimination *in utero*, without any external influences such as tobacco smoke or air pollution. Moreover, we performed all analytic procedures in the same laboratory. We thus believe that hair Pb concentrations can be useful to assess accumulated Pb concentrations from low-dose exposures that were negatively associated to cognitive skills (Huel et al. 1992; Tang et al. 1999; Torrente et al. 2005).

Maternal Hg is a strong predictor of Ca pump activity in all models. The stronger relationship with Hg compared with Pb confirms previous experimental studies suggesting that Hg is a more potent inhibitor of the Ca pump (Viarengo et al. 1993). This statistical association persisted even after adjustment for blood selenium concentrations available for all mother–newborn pairs (data not shown). In newborns, after adjustment for all potential co-factors including Pb exposure and maternal Ca pump activity, only basal Ca pump activity highly correlated with hair Hg. Calmodulin-stimulated activity was not entered into the model, as it was in one *in vitro* study that reported the inhibition of calmodulin-stimulated activity of Ca pump in monkey brain by metal ion exposure (Vig et al. 1989). In study mothers, both stimulated and basal activities correlated with hair Hg without any significant correlation with Pb concentrations. Our finding thus suggests that low-dose Hg exposure could interfere with Ca homeostasis not only in newborns but also in adults.

The independent statistical association of Hg and Pb with Ca pump activity suggests different mechanisms of Ca-mediated toxicity for these heavy metals. Pb was reported to substitute Ca ions and bind to calmodulin (Habermann et al. 1983). MeHg, via its affinity to cellular proteins, disrupts several cascades of Ca^2+^ signaling such as release of Ca^2+^ from intracellular compartments, generates reactive oxygen species, and prevents the formation of phosphorylated intermediates (Gasso et al. 2001; Limke et al. 2003; Viarengo et al. 1993). Extrapolation of results from *in vitro* studies with precisely measured neurotoxicant concentrations to an epidemiologic study using indirect assessment of exposures in humans in blood and hair, such as in our study, is difficult.

In conclusion, there exists an unmet need for biomarkers for epidemiologic study purposes. Our study indicates that a variation in Ca pump activity exists in relation to very low doses of exposure to chemicals interfering with Ca mechanisms of toxicity. One priority for modern environmental toxicology is to bridge experimental approaches and human studies, especially with respect to pollutants present in the daily environment of the human general population. Further experimental research is thus needed to determine any causal link that might underlie observed correlations for Ca-disrupting chemicals at exposure doses relevant to the general population. In addition, the correlation between peripheral biomarkers, such as Ca pump activity, and neurodevelopmental skills should be investigated further to identify the best predictive surrogate biomarkers to use for epidemiologic study purposes in newborns.

## Figures and Tables

**Table 1:** Biological variables in 81 mother–newborn pairs with complete data.

	Arithmetic mean ± SD	Geometric mean	Median	5th Percentile	95th Percentile
Hair Hg (μg/g)					
Mother	1.37 ± 0.94	1.19	1.20	0.54	2.90
Ca pump activity (nmol/mg/hr)					
Mother	2,545 ± 782	2,411	2,559	1,200	3,703
Cord	2,703 ± 674	2,632	2,689	1,613	3,902
Ca pump activity stimulated by calmodulin (nmol/mg/hr)					
Mother	2,963 ± 861	2,824	2,992	1,311	4,312
Cord	3,167 ± 703	3,104	3,114	1,983	4,492
Hair Pb (μg/g)					
Mother	3.63 ± 4.47	3.28	1.80	0.10	9.60
Newborn	1.22 ± 1.41	1.98	0.80	0.10	3.80
Blood Pb (μg/L)					
Mother	60.0 ± 25.3	56.6	54.5	29.5	99.5
Cord	35.4 ± 17.2	32.9	33.0	14.0	62.0

**Table 2:** Multiple regression analysis of Ca pump activities (nmol/mg/hr) in relation to hair Hg and Pb (μg/g).

Variable	Slope estimate ± SE	*t*	*p*	*R*^2^
Ca pump in mother				
HgHM	−0.501 ± 0.183	−2.74	< 0.01	8.1
PbHM	−0.047 ± 0.125	−0.37	0.71	
Ca pump stimulated by calmodulin in mother				
HgHM	−0.470 ± 0.172	−2.73	< 0.01	8.9
PbHM	−0.114 ± 0.118	−0.96	0.34	
Ca pump activity in cord				
HgHM	−0.458 ± 0.095	−4.78	< 0.0001	33.2
PbHC	−0.531 ± 0.122	−4.35	< 0.0001	
Ca pump stimulated by calmodulin in cord				
HgHM	−0.275 ± 0.093	−2.95	< 0.01	11.6
PbHC	−0.179 ± 0.119	−1.51	0.13	

**Table 3:** Multiple regression analysis of Ca pump activities (nmol/mg/hr) in relation to Hg in mother’s hair and Pb content in blood.

Variable	Slope estimate ± SE	*t*	*p*	*R*^2^
Ca pump in mother				
HgHM (μg/g)	−0.423 ± 0.172	−2.45	< 0.05	6.9
PbBM (μg/L)	−0.007 ± 0.235	−0.03	0.97	
Ca pump stimulated by calmodulin in mother				
HgHM (μg/g)	−0.406 ± 0.159	−2.55	< 0.05	7.7
PbBM (μg/L)	−0.048 ± 0.219	−0.22	0.82	
Ca pump activity in cord				
HgHM (μg/g)	−0.398 ± 0.119	−3.35	< 0.01	19.9
PbBC (μg/L)	−0.326 ± 0.135	−2.41	< 0.05	
Ca pump stimulated by calmodulin in cord				
HgHM (μg/g)	−0.285 ± 0.113	−2.52	< 0.01	8.2
PbBC (μg/L)	−0.095 ± 0.128	−0.74	0.46	

**Table 4:** Relationship between Ca pump activities in cord blood (nmol/mg/hr) with Hg and Pb levels adjusted for Ca pump activities in mother.

Variable	Slope estimate ± SE	*t*	*p*	*R*^2^
Ca pump in cord				
HgHM (μg/g)	−0.357 ± 0.105	−3.38	<0.0001	40.2
PbHC (μg/g)	0.209 ± 0.062	3.32	< 0.0001	
CaM (nmol/mg/hr)	−0.369 ± 0.136	−2.71	<0.001	
Ca pump stimulated by calmodulin in cord				
HgHM (μg/g)	−0.214 ± 0.108	−1.97	0.05	23.4
PbHC (μg/g)	0.188 ± 0.067	2.78	<0.001	
CaMc (nmol/mg/hr)	−0.199 ± 0.140	−1.42	0.16	
